# External validation of a new predictive model for falls among inpatients using the official Japanese ADL scale, Bedriddenness ranks: a double-centered prospective cohort study

**DOI:** 10.1186/s12877-022-02871-5

**Published:** 2022-04-15

**Authors:** Masaki Tago, Naoko E. Katsuki, Eiji Nakatani, Midori Tokushima, Akiko Dogomori, Kazumi Mori, Shun Yamashita, Yoshimasa Oda, Shu-ichi Yamashita

**Affiliations:** 1 Department of General Medicine, Saga University Hospital, 5-1-1 Nabeshima, Saga, 849-8501 Japan; 2 Graduate School of Public Health, Shizuoka Graduate University of Public Health, Shizuoka, Japan; 3 Translational Research Center for Medical Innovation, Foundation for Biomedical Research and Innovation at Kobe, Hyogo, Japan; 4 Department of General Medicine, Yuai-Kai Foundation and Oda Hospital, Saga, Japan

**Keywords:** Bedridden, Bedriddenness ranks, Fall, Predictive model, Validation, Saga fall risk models (SFRM)

## Abstract

**Background:**

Several reliable predictive models for falls have been reported, but are too complicated and time-consuming to evaluate. We recently developed a new predictive model using just eight easily-available parameters including the official Japanese activities of daily living scale, Bedriddenness ranks, from the Ministry of Health, Labour and Welfare. This model has not yet been prospectively validated. This study aims to prospectively validate our new predictive model for falls among inpatients admitted to two different hospitals.

**Methods:**

A double-centered prospective cohort study was performed from October 1, 2018, to September 30, 2019 in an acute care hospital and a chronic care hospital. We analyzed data from all adult inpatients, for whom all data required by the predictive model were evaluated and recorded. The eight items required by the predictive model were age, gender, emergency admission, department of admission, use of hypnotic medications, previous falls, independence of eating, and Bedriddenness ranks. The main outcome is in-hospital falls among adult inpatients, and the model was assessed by area under the curve.

**Results:**

A total of 3,551 adult participants were available, who experienced 125 falls (3.5%). The median age (interquartile range) was 78 (66–87) years, 1,701 (47.9%) were men, and the incidence of falls was 2.25 per 1,000 patient-days and 2.06 per 1,000 occupied bed days. The area under the curve of the model was 0.793 (95% confidence interval: 0.761–0.825). The cutoff value was set as − 2.18, making the specificity 90% with the positive predictive value and negative predictive value at 11.4% and 97%.

**Conclusions:**

This double-centered prospective cohort external validation study showed that the new predictive model had excellent validity for falls among inpatients. This reliable and easy-to-use model is therefore recommended for prediction of falls among inpatients, to improve preventive interventions.

**Trial registration:**

UMIN000040103 (2020/04/08)

**Supplementary Information:**

The online version contains supplementary material available at 10.1186/s12877-022-02871-5.

## Background

Falls among inpatients are unfortunate for both patients and their families, caregivers, and wider society [1–5]. Residual pain or fear of recurrence could decrease ability to carry out physical activities or activities of daily living (ADLs) [1, 2], with increased medical costs for additional examinations, treatments, or extended lengths of hospital stay [3, 4]. Inclination to blame medical staff could also result in lawsuits [5]. Many hospitals have therefore tried to assess the risk of falls among inpatients [6]. Several assessment tools, including the Hendrich II Fall Risk Model (HFRM), Morse Fall Scale, and St Thomas Risk Assessment Tool in Falling Elderly Inpatients have been reported to be reliable and useful [7, 8]. However, most Japanese hospitals are reluctant to use them because they are complicated and time-consuming. Instead, they prefer their own easy-to-use, though statistically untried, predictive models for falls [6].

We recently reported two types of new logistic regression models to predict falls of inpatients, named Saga fall risk models (SFRM). These used only simple predictors including Bedriddenness ranks, the official Japanese classifications of ADLs defined by Ministry of Health, Labour and Welfare (MHLW), Japan, easily available and widely used in the medical care system [9]. Even parsimonious Model 2, which used only age and seven factors, showed a high degree of usability. Although our retrospective study showed that the discrimination performance of this model was excellent [9], it has not yet been prospectively validated.

This study therefore examined to assess the discrimination and goodness of fit of one of SFRM, Model 2, for falls among inpatients, through a prospective external validation study at two very different institutions.

## Methods

### Study design, setting, and participants

This was a double-centered prospective cohort study for external validation purposes. We selected the two different types of hospitals to generalize the research findings as much as possible by covering the majority of inpatients in Japan. Acute care hospitals account for 58% of hospital beds in Japan, and convalescent care hospitals account for a further 21% [10]. We included all inpatients aged ≥ 20 years admitted to two different hospitals with different characters, an acute care hospital (Yuai-Kai Foundation and Oda Hospital: Hospital O) and a hospital mainly providing chronic care (Saga City Fuji-Yamato Spa Hospital: Hospital F) (S1, Appendix), between October 2018 and September 2019.

### Data

All the required data were recorded in the electronic medical charts or health records of each hospital. Breakdown of required data extracted from the charts or records were described in S2, Appendix. The departments of admission were categorized into internal medicine, neurosurgery, and others. The attending nurse assessed MHLW Bedriddenness ranks [11, 12], Cognitive function scores [11, 12], Barthel index [13], Katz index [14], use of hypnotic medications [15], permanent residual damage from previous stroke [16, 17], history of falls [16, 18], and visual impairment [11] of all inpatients aged 20 years or older in the course of regular clinical care within 72 h after admission. Bedriddenness ranks classified into five grades (normal, J: independence/autonomy, A: housebound, B: chair-bound, or C: bed-bound), and Cognitive function scores into six grades (normal, I, II, III, IV, and M) [11, 12], both of which are official Japanese classifications of ADLs, widely used in Japanese health insurance and nursing care insurance systems. Mini-Mental State Examination (MMSE) [19], and ABC dementia scale [20] were assessed by attending nurses or research assistants within 72 h of admission, for those determined to have an abnormal Cognitive function score by the attending nurse on admission. High inter-rater reliability and criterion-related validity of Cognitive function scores were shown in our previous study [12], and MMSE and ABC-DS are well-established and objective scales for dementia [19, 20]. Hypnotic medications included benzodiazepines and non-benzodiazepines, except for melatonin receptor agonists and orexin receptor antagonists, in line with a previous study [9]. Permanent residual damage from previous stroke was defined as paralysis of the lower limbs of any severity caused by cerebral infarction, cerebral hemorrhage, or subarachnoid hemorrhage. A history of falls was defined as a fall within the year before admission, regardless of the presence or absence of trauma or site of occurrence, inside or outside the hospital. Visual impairment was defined as binocular vision < 20/40 (vision) by near-vision test, with or without visual correction. The vision test was performed in precisely the same way at both hospitals using the same tool placed at the same distance from the individual patient. This made it possible to assume that the test had adequate reliability and reproducibility. The attending nurse or nurse witnessing a fall event recorded the incident in a fall-specific report form as part of an incident/accident report as soon as practicable. The sources of incident/accident records for falls used in this study were both daily incident reports and reports specially created for the research. Falls were defined as an unexpected fall with or without injury, from any height or position i.e., from stairs, chair, or bed, when standing, walking, sitting, or recumbent [9]. The primary condition causing the admission was registered by the attending physician following the International Statistical Classification of Diseases and Related Health Problems Tenth Revision (ICD-10).

Comprehensive fall prevention strategies such as providing instructions on footwear, fall preventive movement sensors or monitoring cameras, restraining belts, bed rails, or aiding transfer to the toilet, or fall injury reduction strategies, such as impact-absorbing mats or low beds, were used following an assessment of risk with existing assessment tools individually developed by each hospital. We did not tell attending nurses and physicians the results of our model’s predictions.

### Statistical analysis

We chose Model 2 (S3, Appendix) from two models developed, because it requires fewer items than Model 1 [9]. Continuous and categorical variables were shown as median (interquartile range) and absolute number (percentage). Those variables were separately derived from all patients both with and without falls, with falls, or without falls, and for those admitted to both hospitals, and hospitals O and F separately. Multiplicity was not adjusted to control type I errors in the exploratory analysis. Multivariate logistic regression analysis used all eight factors required by Model 2: age, sex, emergency admission, department of admission, use of hypnotic medications, history of falls, independence of eating, which is one of the assessment items of the Barthel index, and MHLW Bedriddenness ranks [9]. We also calculated fall probability, sensitivity, specificity, positive predictive value (PPV), and negative predictive value (NPV) for each cutoff value derived from a sensitivity of 90%, the Youden index, or specificity of 90% of the predictive model in this study, and for that set by our previous study [9]. We assessed the predictive performance of the model by calculating AUC, 95% confidence interval (CI), and shrinkage coefficient of patients admitted to each hospital and overall. In the subgroup analysis, we compared AUCs and 95% CIs between two groups of patients by factors other than the eight items above, i.e., hospital of admission, undergoing a surgical operation, rehabilitation, ambulance transport, admission with a referral letter from a primary physician, MHLW Cognitive function scores (normal, I, II, III, IV, M), permanent residual damage from previous stroke, visual impairment, and a hospital stay of at least the median length (≥ 10 days). Analyses used SPSS statistics version 27 IBM.

### Sample size

We determined the sample size of 775 patients based on the effect size of 0.20 (predicted AUC of 0.70, null hypothesis AUC of 0.50), a fall rate of 4.0%, alpha error of 0.05 and beta error of 0.20, estimated by the AUC of 0.787 reported in a previous study [9].

### Ethical considerations

This study conforms to the ethical guidelines for medical and health research involving human subjects issued by the MHLW and the Ministry of Education, Culture, Sports, Science, and Technology in Japan. This study was approved by the research ethics committee of the Yuai-Kai Foundation and Oda Hospital (No. 20180629). The study was registered at the University Hospital Medical Information Network (UMIN) at www.umin.ac.jp (UMIN ID: UMIN000040103). We obtained consent from all patients using the hospital’s comprehensive agreement method, and anonymity of patients was protected.

## Results

### Patients’ background and incidence of falls

**Fig. 1:**
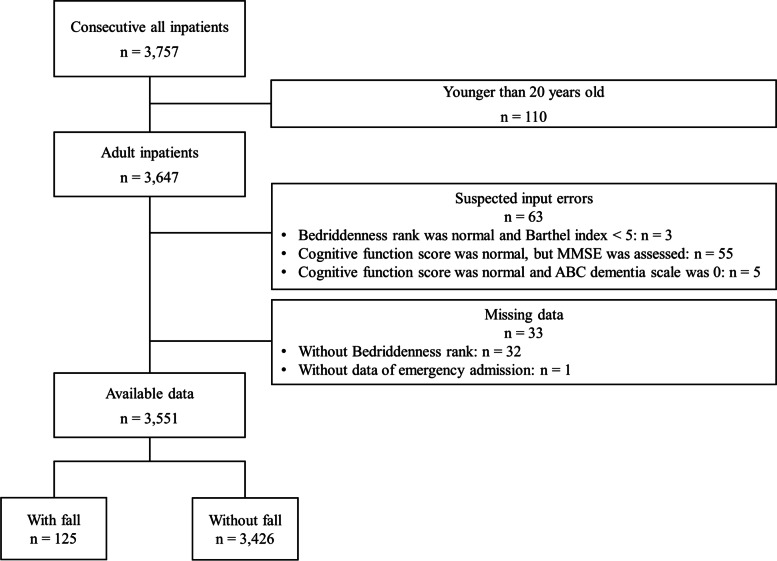
Data flow diagram. A total of 3,551 adult participants were eligible, who experienced a total of 125 falls (3.5%)

During the study period, 3,757 inpatients were admitted, of whom 110 were excluded because they were under 20 years old, 63 because of input errors (MHLW Bedriddenness rank was normal despite Barthel index < 5; MHLW Cognitive function score was normal, but MMSE was assessed; Cognitive function score was normal despite ABC dementia scale score of 0), and 33 because of lack of information. This gave a total of 3,551 eligible cases (Fig. 1). In total, 125 falls occurred in this group (3.5%), the median age (interquartile range) was 78 (66–87) years, 1,701 (47.9%) were men, the median length of hospital stay (interquartile range) was 10 (5–18) days, and the incidence of falls was 2.25 per 1,000 patient-days and 2.06 per 1,000 occupied bed days (Table 1). The incidence of falls by age groups and classification of diseases causing emergency or scheduled admissions to each hospital are shown in the supplementary files (S4, Figure and S5, Table).

**Table 1:** Characteristics of patients and the results of univariate analysis

**Variable, Category**	**All patients** ***n* = 3,551**	**With Fall**	**Without fall**	***p*** **value**^**†**^
		***n* = 125**	***n* = 3,426**	
Age, years	78 (66–87)	86 (80–90)	78 (65–86)	< 0.001
Gender, Male	1,701 (47.9)	59 (47.2)	1,642 (47.9)	0.873
Emergency admission, Yes	1,374 (38.7)	77 (61.6)	1,297 (37.9)	< 0.001
Transported by ambulance, Yes	473 (13.3)	15 (12.0)	458 (13.4)	0.658
Referral letter, Presence	1,064 (30.0)	43 (34.4)	1,021 (29.8)	0.324
Department, Internal Medicine	1,999 (56.3)	89 (71.2)	1910 (55.8)	< 0.001
Department, Neurosurgery	68 (1.9)	7 (5.6)	61 (1.8)	
Hypnotic medications, Using	357 (10.1)	28 (22.4)	329 (9.6)	< 0.001
Hypnotic medications, Missing	7 (0.2)	0 (0)	7 (0.2)	
Permanent residual damage from previous stroke, Presence	227 (6.4)	11 (8.8)	216 (6.3)	0.273
History of falls, Presence	352 (10.0)	43 (34.4)	309 (9.1)	< 0.001
Visual impairment, Presence	1,135 (34.2)	61 (56.0)	1,074 (33.5)	< 0.001
Eating, Independent	2,713 (76.4)	66 (52.8)	2,647 (77.3)	< 0.001
Eating, Missing category	45 (1.2)	0 (0)	45 (1.3)	
Bedriddenness rank, Normal	1,790 (51.9)	11 (8.8)	1,779 (51.9)	< 0.001
Bedriddenness rank, J	317 (8.9)	10 (8.0)	307 (9.0)	
Bedriddenness rank, A	410 (11.5)	25 (20.0)	385 (11.2)	
Bedriddenness rank, B	517 (14.6)	57 (45.6)	460 (13.4)	
Bedriddenness rank, C	517 (14.6)	22 (17.6)	495 (14.4)	
Cognitive function score, Normal	2,187 (61.6)	20 (16.1)	2,167 (63.3)	< 0.001
Cognitive function score, I	423 (11.9)	24 (19.4)	399 (11.7)	
Cognitive function score, II	341 (9.6)	35 (28.2)	306 (8.9)	
Cognitive function score, III	453 (12.8)	35 (28.2)	418 (12.2)	
Cognitive function score, IV	119 (3.4)	8 (6.5)	111 (3.2)	
Cognitive function score, M	16 (0.5)	2 (1.6)	14 (0.4)	
Cognitive function score, missing	9 (0.3)	0 (0)	9 (0.3)	
Barthel index	100 (55–100)	50 (25–65)	100 (55–100)	< 0.001
Katz index	6 (1–6)	1 (0–3)	6 (1–6)	< 0.001
Surgical operation, Undergone	991 (27.9)	13 (10.4)	978 (28.5)	< 0.001
Rehabilitation, Undergone	1,310 (36.9)	85 (68.0)	1225 (35.8)	< 0.001
Length of hospital stay (days)	10 (5–18)	32 (18–56)	9 (5–17)	< 0.001

Continuous and categorical variables are shown as median (interquartile range) and frequency (percent). Bedriddenness ranks: J, independence/autonomy; A, house-bound; B, chair-bound; C, bed-bound. Cognitive function scores: I, almost independent in daily living with only slight cognitive impairment; II, independent with slight difficulty in daily living or communication under careful overseeing; III, dependent in daily living or communication; IV, dependent in daily living or communication, and requires constant care; M, severe psychological symptoms, troubled behaviors or severe physical disorders requiring specialized medical service

^†^*p* values were calculated by Mann–Whitney U-test for continuous variables and chi-squared test for categorical variables

### Univariate analysis

The results of the univariate analysis are shown in Table 1. The patients who fell were significantly older (86 years vs. 78 years), and had significantly longer hospital stay (32 days vs. 9 days), significantly lower Barthel Index (50 vs. 100), significantly higher rates of emergency admission (61.6% vs. 37.9%), and greater use of hypnotic medications (22.4% vs. 9.6%), were more likely to have a history of falls (34.4% vs. 9.1%), visual impairment (56.0% vs. 33.5%), and be undergoing rehabilitation (68.0% vs. 35.8%), and were significantly less likely to be eating independently (52.8% vs. 77.3%) and undergoing surgery (10.4% vs. 28.5%). A greater proportion of patients in the group who fell were admitted to the Department of Internal Medicine or Neurosurgery, had Bedriddenness ranks of A, B, or C, and Cognitive function scores of I, II, III, IV, or M, with significantly different distributions from those who did not have a fall. There were no significant differences between the groups in sex, use of ambulance transport, admission with a referral letter from a primary physician, or permanent residual damage from previous stroke.

### Multivariate analysis and performance of predictive models

**Fig. 2:**
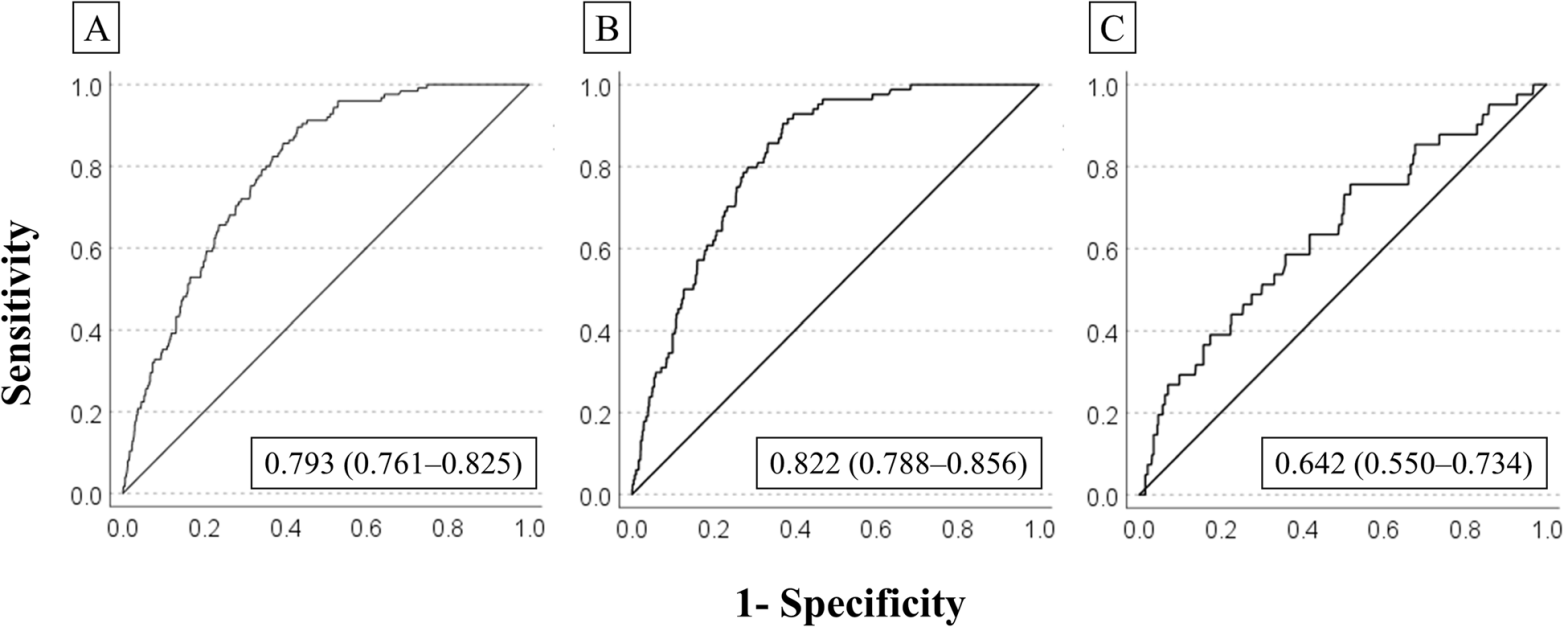
Receiver operating characteristics (ROCs) and areas under the curves (AUCs). ROC of the predictive model for falls for all patients (**A**), and for patients in Hospital O (**B**), and Hospital F (**C**)

**Fig. 3:**
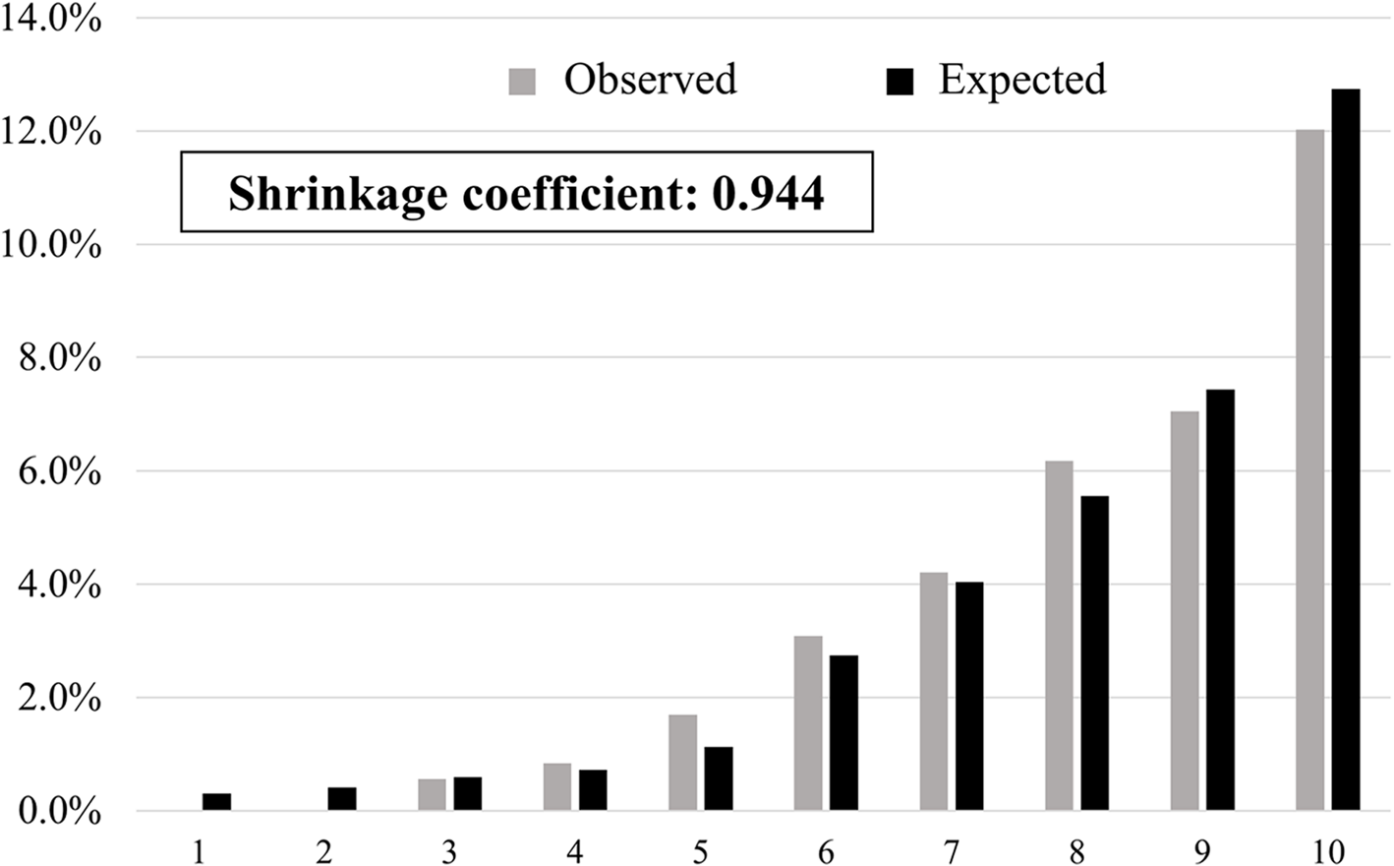
The predicted and observed rates of falls in 10 groups divided into 10 deciles by score using the predictive model. The gap between the predicted and observed values was small enough to use in prediction of falls for either group, with excellent calibration of the model

The multivariate logistic regression analysis showed a statistically significant relationship between falls and admission to the Department of Neurosurgery, history of falls, and Bedriddenness ranks (Table 2). The performance of the predictive model, measured as AUC, was 0.793 (95% CI: 0.761–0.825) (Fig. 2-A). The cutoff values for the predictive model with a sensitivity of 90%, the Youden index, and specificity of 90% were − 3.26, − 3.17, and − 2.18, respectively. The PPV and NPV derived from each cutoff point were 7.0% and 99%, 7.1% and 99%, and 11.4% and 97%, respectively (Table 3). We also examined the validation results using the cutoff values of the previous study [9] in supplementary Table (S6, Table). The incidence of falls actually observed was consistent with the predicted incidence calculated using the predictive model, with a shrinkage coefficient of 0.944 (Fig. 3).

**Table 2:** Results of multivariate logistic regression analysis

**Variable, Category (Reference)**	**OR**	**95% CI**	***p*** **value**^**†**^
Age	1.0	1.0–1.0	0.105
Gender, Male (Female)	1.3	0.9–1.8	0.226
Emergency admission, Presence (Absence)	1.4	0.9–2.0	0.138
Department, Internal Medicine (Others)	1.1	0.7–1.7	0.831
Department, Neurosurgery (Others)	2.9	1.1–7.2	0.026
Hypnotic medications, Using (Not using)	1.5	0.9–2.4	0.084
Hypnotic medications, Missing (Not using)	0.0	0.0	1.000
History of falls, Presence (Absence)	2.5	1.7–3.7	0.000
Eating, Independent (Requiring assistance)	0.9	0.6–1.4	0.561
Eating, Missing category (Requiring assistance)	0.0	0.0	0.999
Bedriddenness rank, J (Normal)	3.3	1.3–8.2	< 0.001
Bedriddenness rank, A (Normal)	5.6	2.5–12.3	< 0.001
Bedriddenness rank, B (Normal)	9.4	4.3–20.6	< 0.001
Bedriddenness rank, C (Normal)	3.4	1.4–8.5	< 0.001

Bedriddenness ranks: J, independence/autonomy; A, house-bound; B, chair-bound; C, bed-bound

^†^*p* values for Wald test

**Table 3:**
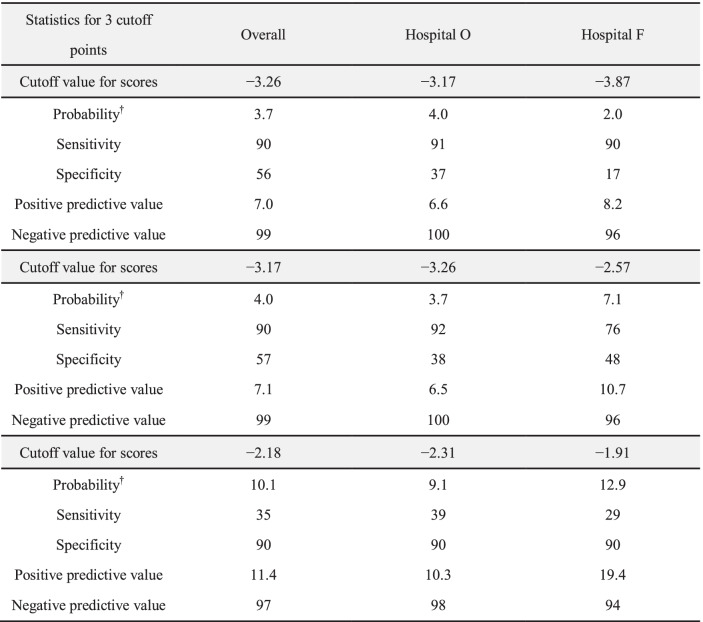
Validation of the predictive model with the cutoff points determined in the present study

^†^The value was calculated as the probability of a fall for patients with defined score

Three cutoff points were determined by the minimum score over 90% sensitivity, the optimal point by Youden index, and the maximum score over 90% specificity

The sensitivity, specificity, positive predictive value, and negative predictive value of the model derived from overall patients were 90%, 56%, 7.0%, and 99% with the cutoff score of − 3.26, 90%, 57%, 7.1%, and 99% with − 3.17, and 35%, 90%, 11.4%, and 97% with − 2.18, respectively. In similar fashion, the sensitivity and specificity of the model derived from Hospital O were 91%, 37%, 6.6%, and 100% with the cutoff score of − 3.17, 92%, 38%, 6.5%, and 100% with − 3.26, and 39%, 90%, 10.3%, and 98% with − 2.31, respectively. The sensitivity and specificity of the model derived from Hospital F were 90%, 17%, 8.2%, and 96% with the cutoff score of − 3.87, 76%, 48%, 10.7%, and 96% with − 2.57, and 29%, 90%, 19.4%, and 94% with − 1.91, respectively

In the analysis by age group, the AUCs of the predictive model were 0.941 (0.891–0.992) for those younger than 65 years old, 0.668 (0.480–0.856) for those aged 65 to 69 years old, 0.730 (0.588–0.873) for those aged 70 to 74 years old, 0.834 (0.732–0.936) for those aged 75 to 79 years old, 0.784 (0.690–0.879) for those aged 80 to 84 years old, 0.687 (0.601–0.772) for those aged 85 to 89 years, and 0.668 (0.586–0.749) for those aged 90 years or older (S7, Figure).

### Subgroup analysis

Background characteristics of each hospital and univariate analysis of each item between patients in the two groups are shown in S8, Table. In Hospital O, 2,970 inpatients were eligible, with 84 suffering falls (2.8%). The median age (interquartile range) of all the patients was 77 (64–86) years, 48.2% were men, and the median length of hospital stay (interquartile range) was 9 (4–16) days. In Hospital F, 581 inpatients were eligible, with 41 suffering falls (7.0%). The median age (interquartile range) of all the patients was 84 (74–90) years, 46.5% were men, and the median length of hospital stay (interquartile range) was 17 (8–38) days.

The results of the univariate analysis of those from Hospital O were similar to those among all patients, except that a significantly higher percentage of patients who fell were admitted with a referral letter from a primary physician (45.2% vs. 34.3%). In Hospital F, there were no significant differences among the variables including age, emergency admission, use of hypnotic medications, visual impairment, independence of eating, Barthel Index, or Katz Index, which showed significant differences when calculated for all patients.

The performance of the predictive model measured as AUC was 0.822 (95% CI: 0.788–0.856) for those in Hospital O. However, the AUC for several subgroups was less than 0.7. This included those from Hospital F (0.624; 95% CI: 0.550–0.734), transported by ambulance (0.694; 95% CI: 0.574–0.813), with a Cognitive function score of I (0.653; 95% CI: 0.547–0.759), II (0.676; 95% CI: 0.583–0.769), III (0.587; 95% CI: 0.491–0.683), or IV (0.641; 95% CI: 0.426–0.856), and whose hospital stay was ≥ 10 days (0.694; 95% CI: 0.648–0.740) (Fig. 2-B, C, and S9, Figure).

## Discussion

This study was a prospective external validation of the predictive model for falls of inpatients recently developed and reported in a previous study [9]. The discrimination power of the model was high, shown by AUC of 0.793 (95% CI: 0.761–0.825) (Fig. 2-A). This was comparable with the figures for previous predictive models for falls such as the HFRM, Morse Fall Scale, and St Thomas Risk Assessment Tool in Falling Elderly Inpatients, ranging 0.71–0.80 [21]. The shrinkage coefficient was 0.944 (Fig. 3), showing excellent calibration. We designed our predictive model to evaluate all patients on admission to predict the risk of in-hospital falls. However, there are limited medical resources for fall prevention, and it is therefore essential to focus on the highest-risk group for falls, most of whom would naturally be the oldest patients. We therefore set the cutoff value as − 2.18 in this study, making the specificity 90% with PPV and NPV at 11.4% and 97%. These values were comparable to those of a previous study [9], indicating the reliability of this predictive model. The most significant characteristics of our model are its simplicity and usability. It requires only eight assessment items, which is similar or fewer than the number required by alternative reliable models [7, 8]. Six of the evaluation items are mandatory information routinely collected on admission in Japan. The two other items, use of hypnotic medications and history of falls, are readily available, which makes the model much simpler and easier to use [7, 8]. In addition, it is easy to put the equation into the electronic medical record system, or into Excel software on a personal computer, which makes the model readily available at patients’ bedsides.

Admission to the Department of Neurosurgery, a history of falls, and Bedriddenness ranks showed a significant relationship with falls in the multivariate logistic regression analysis. However, items that showed a significant relationship in a previous study (being male, using hypnotic medications, and a group with missing data on eating) [9], all failed to show any relationship (Table 2). Being female had previously been reported as a risk [15], so the background of the study population could have affected the result. On use of hypnotic medications, we did not include newly available melatonin receptor agonists or orexin receptor antagonists [22], in line with our previous study [9]. However, we assumed that these drugs would not affect the risk of falls significantly, because the new drugs have a much lower incidence of side effects such as lightheadedness [23]. Contrary to our previous retrospective study [9], this study accurately recorded ADLs, including ability to eat, for almost all patients, because it was a prospective study, resulting in the absence of a significant relationship between eating ability and falls (Table 2). Most importantly, this study also showed MHLW Bedriddenness ranks had a high odds ratio and strong association with falls. Bedriddenness ranks was the key item in this model. This study reconfirms the usefulness and reliability of MHLW Bedriddenness ranks.

Subgroup analysis showed an AUC of slightly less than 0.7 in the groups transported by ambulance, with Cognitive function score of I, II, III, and IV, admitted to Hospital F, and with length of hospital stay ≥ 10 days (S9, Figure). Ambulance transport was not included in the model’s assessment items, but it showed a moderate correlation with emergency admission. Similarly, the level of Cognitive function scores, which were not included in the model, showed a moderate correlation with included Bedriddenness ranks. This might have resulted in the lower usefulness in predicting falls of emergency admissions in the group of patients transported by ambulance and of Bedriddenness ranks in the groups with Cognitive function scores of I, II, III, and IV. Additionally, tending to wander around could be one of risk factors of falls [24–26]. However, our model showed enough reliability without the item. It is also possible that the discrimination of the model could be further improved with the addition of measurement of cognitive impairment, because this study also included 38.2% of patients with dementia [15, 27, 28]. Conversely, the model showed good discrimination for surgical operations, rehabilitation, admission with a referral letter from a primary physician, and permanent residual damage from previous stroke, with AUC ranging from 0.708 to 0.895, which would defy the necessity of these items in the prediction.

Contrary to Hospital O with an AUC of 0.822, the group admitted to Hospital F, which mainly provides chronic care, had an AUC of 0.642. Hospital F group had a higher percentage of patients who were older and showed cognitive impairment. The primary conditions associated with both scheduled and emergency admissions were very different between the two hospitals. Those with Cognitive function scores of I, II, III, and IV also had AUCs less than 0.7, mildly lowering the discrimination of this model. This suggests that the higher proportion of patients with cognitive impairment may be one of the reasons for the lower AUC in Hospital F. Unlike Hospital O, Age and percentage of emergency admissions showed no significant differences in Hospital F. Despite a natural assumption, the oldest patients could have lower risk because of inactivity or immobility [29, 30]. This suggestion is supported by the fact that the fall rate was lower among Bedriddenness rank C than A or B, and that the peak fall rate was between the ages of 86 and 90, with a subsequent decrease (S4, Figure) [9]. Having more older patients at Hospital F could therefore have prevented the fall risk from increasing or even reduced it. It is reasonable to expect that patients with emergency admissions are generally more unstable [9, 31]. However, discrepancy in the definition of emergency and scheduled admissions between the two hospitals could mean that this assumption was not applicable. There was considerable variation in the primary conditions causing scheduled admissions in Hospital O. However, the list was more limited in Hospital F, and included colorectal polyps (6.6%), trochanteric fracture of the femur (5.7%), complications to permanent residual damage from previous stroke (4.7%), and aspiration pneumonia (4.7%) (S5, Table). Patients with femur fractures may have been listed as those with a history of falls, because more than 95% of femur fractures were caused by falls [32]. Permanent residual damage from previous stroke was also one of the fall risks [33]. Aspiration pneumonia could be unsuitable for comparison in Hospital F, because it was listed as a causative condition of both scheduled and emergency admissions (S5, Table).

The level of the assessment items for the predictive model could change during hospitalization [34]. For example, emergency admission may be related to other fall risk factors such as fever, dehydration, arrhythmia, or dizziness [35, 36]. These fall risk factors can improve quickly or even disappear during a long hospital stay. A patient without a history of falls on admission could suffer a fall during hospitalization. It is also common for hypnotic medications to be newly prescribed, which could markedly increase fall risk [37]. Regular reassessment of fall risk should therefore be considered, especially during prolonged hospitalization.

### Limitations

A major limitation of this prospective study was that many of the patients may have come from similar backgrounds, because we included a high percentage of participants from Hospital O, which is also where the model was developed, albeit with different patients. It is therefore essential to carry out further validation work involving more hospitals and facilities of different types. It is also desirable to develop other predictive models for falls that could be suitable for different types of hospitals, or consider changes in patients’ condition during hospitalization. The data on falls in this study were derived from both the daily incident reporting system and reports specially created for the research purpose. However, the incidence of falls could have been underreported [38, 39], which would have affected the results. In addition, the two hospitals had implemented comprehensive fall prevention measures [40–42] with established evidence in this study, even though many fall prevention measures lacked established evidence. This could therefore have influenced the study results. Furthermore, it is desirable to prospectively validate if this predictive model has actual effect of reducing the incident of fall or fall-related injury among inpatients, before justifying the routine use of it. We did not compare the accuracy of the study predictive model with other existing predictive models for falls in the population of this study.

## Conclusion

External validation in a double-centered prospective cohort study found that the predictive model for falls among inpatients developed in a previous study showed excellent discrimination. We recommend this reliable and easy-to-use model to predict falls among inpatients and provide effective preventive interventions.

## Supplementary Information

**Additional file 1: Appendix S1.** Characteristics of the two hospitals.

**Additional file 2: Appendix S2.** Breakdown of required data.

**Additional file 3: Appendix** **S3.** The formula of model 2.

**Additional file 4: Figure S4.** The incidence of falls among inpatients, by age group

**Additional file 5: Table S5.** Classification of primary condition causing emergency or scheduled admission to hospital.

**Additional file 6: Table S6.** Validation of the predictive model with the cutoff points of the previous study.

**Additional file 7: Figure S7.** The results of the analysis by age group

**Additional file 8: Table S8. **Patient backgrounds and results of univariate analysis by hospital.

**Additional file 9: Figure S9.** The results of subgroup analysis.

## Data Availability

The datasets generated and analyzed during the current study are available in the UMIN-ICDR repository, https://upload.umin.ac.jp/cgi-bin/ctr_e/ctr_view.cgi?recptno=R000045659

## Abbreviations

ADLs: Activities of daily living
HFRM: Hendrich II Fall Risk Model
SFRM: Saga fall risk models
MHLW: Ministry of Health, Labour and Welfare
AUC: Area under the curve
MMSE: Mini-Mental State Examination
ICD-10: International Statistical Classification of Diseases and Related Health Problems Tenth Revision
CI: Confidence interval
PPV: Positive predictive value
NPV: Negative predictive value

## References

[1] Choi K, Jeon GS, Cho SI. Prospective Study on the Impact of Fear of Falling on Functional Decline among Community Dwelling Elderly Women. Int J Environ Res Public Health. 2017;14(5). Epub 2017/04/28. 10.3390/ijerph14050469 10.3390/ijerph14050469 PMC5451920 28448461

[2] Deshpande N, Metter EJ, Lauretani F (2008). Activity restriction induced by fear of falling and objective and subjective measures of physical function: a prospective cohort study. J Am Geriatr Soc.

[3] Okamura T (2006). Institutional liability for patients' falls in health care facilities. IRYO -Japanese J Natl Med Serv.

[4] Burns ER, Stevens JA, Lee R (2016). The direct costs of fatal and non-fatal falls among older adults—United States. J Safety Res.

[5] Japan CO (2017). The Aging Society: Current Situation and Implementation Measures. Annual Report on the Aging Society (Summary).

[6] Soyano A, Suzuki M, Harada A, Okada S, Kaminai T (2018). Fall Risk Assessment Tool Usage and Issues: Results of a Survey of Japanese Society for Fall Prevention Members. Japanese Journal of Fall Prevention.

[7] Hendrich AL, Bender PS, Nyhuis A (2003). Validation of the Hendrich II Fall Risk Model: a large concurrent case/control study of hospitalized patients. Appl Nurs Res.

[8] Morse JM, Black C, Oberle K, Donahue P (1989). A prospective study to identify the fall-prone patient. Soc Sci Med.

[9] Tago M, Katsuki NE, Oda Y (2020). New predictive models for falls among inpatients using public ADL scale in Japan: A retrospective observational study of 7,858 patients in acute care setting. PLoS ONE.

[10] Ministry of Health, Labour and Welfare. 2020. Annual Health, Labour and Welfare Report 2020 (Summary). Accessed 8 Jan 2022. https://www.mhlw.go.jp/english/wp/wp-hw13/dl/02e.pdf

[11] Aihara H, Tago M, Oishi T, Katsuki NE, Yamashita SI (2018). Visual impairment, partially dependent ADL and extremely old age could be predictors for severe fall injuries in acute care settings. Int J Gerontol.

[12] Tago M, Katsuki NE, Yaita S, Nakatani E, Yamashita S, Oda Y, Yamashita SI (2021). High inter-rater reliability of Japanese bedriddenness ranks and cognitive function scores: a hospital-based prospective observational study. BMC Geriatr.

[13] Fi M, Dw B (1965). Functional evaluation: the Barthel index. Md State Med J.

[14] Katz S, Ford AB, Moskowitz RW, Jackson BA, Jaffe MW (1963). Studies of illness in the aged: the index of ADL: a standardized measure of biological and psychosocial function. JAMA.

[15] Sousa LM, Marques-Vieira CM, Caldevilla MN, Henriques CM, Severino SS, Caldeira SM (2017). Risk for falls among community-dwelling older people: systematic literature review. Rev Gaucha Enferm..

[16] Ganz DA, Bao Y, Shekelle PG, Rubenstein LZ (2007). Will my patient fall?. JAMA.

[17] Lukaszyk C, Harvey L, Sherrington C, Keay L, Tiedemann A, Coombes J, Clemson L, Ivers R (2016). Risk factors, incidence, consequences and prevention strategies for falls and fall-injury within older indigenous populations: a systematic review. Aust N Z J Public Health.

[18] Graafmans WC, Ooms ME, Hofstee HM, Bezemer PD, Bouter LM, Lips P (1996). Falls in the elderly: a prospective study of risk factors and risk profiles. Am J Epidemiol.

[19] Folstein MF, Folstein SE, McHugh PR (1975). "Mini-mental state". A practical method for grading the cognitive state of patients for the clinician. J Psychiatr Res..

[20] Umeda-Kameyama Y, Mori T, Wada-Isoe K (2019). Development of a novel convenient Alzheimer's disease assessment scale, the ABC Dementia Scale, using item response theory. Geriatr Gerontol Int.

[21] Kim EA, Mordiffi SZ, Bee WH (2007). Evaluation of three fall-risk assessment tools in an acute care setting. J Adv Nurs.

[22] Hoyer D, Allen A, Jacobson LH (2020). Hypnotics with novel modes of action. Br J Clin Pharmacol.

[23] Torii H, Ando M, Tomita H, Kobaru T (2020). Association of Hypnotic Drug Use with Fall Incidents in Hospitalized Elderly Patients: A Case-Crossover Study. Biol Pharm Bull.

[24] Berry SD, Zullo AR, Lee Y (2018). Fracture Risk Assessment in Long-term Care (FRAiL): Development and Validation of a Prediction Model. J Gerontol A Biol Sci Med Sci.

[25] Klein DA, Steinberg M, Galik E (1999). Wandering behaviour in community-residing persons with dementia. Int J Geriatr Psychiatry.

[26] Hendlmeier I, Bickel H, Hessler JB (2018). Demenzsensible Versorgungsangebote im Allgemeinkrankenhaus : Repräsentative Ergebnisse aus der General Hospital Study (GHoSt) [Dementia friendly care services in general hospitals : Representative results of the general hospital study (GHoSt)]. Z Gerontol Geriatr..

[27] Chu LW, Chi I, Chiu AY (2005). Incidence and predictors of falls in the chinese elderly. Ann Acad Med Singap..

[28] Segev-Jacubovski O, Herman T, Yogev-Seligmann G (2011). The interplay between gait, falls and cognition: can cognitive therapy reduce fall risk?. Expert Rev Neurother.

[29] Demura S, Sato S, Minami M (2003). Gender and age differences in basic ADL ability on the elderly: comparison between the independent and the dependent elderly. J Physiol Anthropol Appl Human Sci.

[30] Hou C, Ping Z, Yang K (2018). Trends of Activities of Daily Living Disability Situation and Association with Chronic Conditions among Elderly Aged 80 Years and Over in China. J Nutr Health Aging.

[31] Jung H, Park HA, Hwang H (2020). Improving Prediction of Fall Risk Using Electronic Health Record Data With Various Types and Sources at Multiple Times. Comput Inform Nurs.

[32] Parkkari J, Kannus P, Palvanen M, Natri A, Vainio J, Aho H, Vuori I, Järvinen M (1999). Majority of hip fractures occur as a result of a fall and impact on the greater trochanter of the femur: a prospective controlled hip fracture study with 206 consecutive patients. Calcif Tissue Int.

[33] Srikanth V, Beare R, Blizzard L (2009). Cerebral white matter lesions, gait, and the risk of incident falls: a prospective population-based study. Stroke.

[34] van Seben R, Reichardt LA, Aarden JJ (2019). Hospital-ADL study group. The course of geriatric syndromes in acutely hospitalized older adults: The Hospital-ADL Study. J Am Med Dir Assoc..

[35] Cao B, Ren LL, Zhao F (2010). Viral and Mycoplasma pneumoniae community-acquired pneumonia and novel clinical outcome evaluation in ambulatory adult patients in China. Eur J Clin Microbiol Infect Dis..

[36] Mombelli G, Pezzoli R, Pinoja-Lutz G (1999). Oral vs intravenous ciprofloxacin in the initial empirical management of severe pyelonephritis or complicated urinary tract infections: a prospective randomized clinical trial. Arch Intern Med.

[37] Frighetto L, Marra C, Bandali S (2004). An assessment of quality of sleep and the use of drugs with sedating properties in hospitalized adult patients. Health Qual Life Outcomes.

[38] Evans SM, Berry JG, Smith BJ (2006). Attitudes and barriers to incident reporting: a collaborative hospital study. Qual Saf Health Care.

[39] Kurihara M, Nagao Y, Tokuda Y (2021). Incident reporting among physicians-in-training in Japan: A national survey. J Gen Fam Med.

[40] Oliver D, Connelly JB, Victor CR (2007). Strategies to prevent falls and fractures in hospitals and care homes and effect of cognitive impairment: systematic review and meta-analyses. BMJ..

[41] Tinetti ME, Baker DI, McAvay G (1994). A multifactorial intervention to reduce the risk of falling among elderly people living in the community. N Engl J Med.

[42] Haines TP, Bennell KL, Osborne RH (2004). Effectiveness of targeted falls prevention programme in subacute hospital setting: randomised controlled trial. BMJ.

