# Correction: External validation of a new predictive model for falls among inpatients using the official Japanese ADL scale, Bedriddenness ranks: a double-centered prospective cohort study

**DOI:** 10.1186/s12877-022-03291-1

**Published:** 2022-07-27

**Authors:** Masaki Tago, Naoko E. Katsuki, Eiji Nakatani, Midori Tokushima, Akiko Dogomori, Kazumi Mori, Shun Yamashita, Yoshimasa Oda, Shu-ichi Yamashita

**Affiliations:** 1 Department of General Medicine, Saga University Hospital, 5-1-1 Nabeshima, Saga, 849-8501 Japan; 2 Graduate School of Public Health, Shizuoka Graduate University of Public Health, Shizuoka, Japan; 3 Translational Research Center for Medical Innovation, Foundation for Biomedical Research and Innovation at Kobe, Hyogo, Japan; 4 Department of General Medicine, Yuai-Kai Foundation and Oda Hospital, Saga, Japan

**Correction: BMC Geriatrics 22, 331 (2022)**

**https://doi.org/10.1186/s12877-022-02871-5**

After publication of this article [1], the authors reported that there was a mistake in the formula of model 2 in Additional file 3. The correct formula of model 2 is as follows:

$$-5.8563+0.0096\times \left(\mathrm{Age}\right)+\left(\mathrm{Male}=0.5684\right)+\left(\mathrm{Emergency}\ \mathrm{admission}=0.4418\right)+\left(\mathrm{Admitted}\ \mathrm{department};\;\mathrm{Neurosurgery}=0.6520\right)+\left(\mathrm{Hypnotics};\;\mathrm{Using}=0.3612,\mathrm{Missing}\ \mathrm{data}=0.2139\right)+\left(\mathrm{History}\ \mathrm{of}\ \mathrm{fall}=0.4362\right)+\left(\mathrm{Ability}\ \mathrm{of}\ \mathrm{eating};\;\mathrm{Independent}=0.2352,\mathrm{Missing}\ \mathrm{data}=-1.0436\right)+\left(\mathrm{Bedriddenness} \operatorname {rank};\;\mathrm{J}=1.3758,\mathrm{A}=1.8317,\mathrm{B}=1.9186,\mathrm{C}=1.7205,\mathrm{Not}\ \mathrm{assessable}=-0.1505\right)$$

The original article [1] has been corrected.
