# Supplementary material for: External validation of a new predictive model for falls among inpatients using the official Japanese ADL scale, Bedriddenness ranks: a double-centered prospective cohort study
Source: BMC Geriatr. 2022 Apr 15;22:331. doi: 10.1186/s12877-022-02871-5 (PMC9013105; doi:10.1186/s12877-022-02871-5)
Supplement: Supplementary file 1 — Additional file 1: Appendix S1. Characteristics of the two hospitals. [file 12877_2022_2871_MOESM1_ESM.docx]

External validation of a new predictive model for falls among inpatients using the official Japanese ADL scale, Bedriddenness ranks: A double-centered prospective cohort study

Masaki Tago, MD, PhD^1^*; Naoko E. Katsuki, MD, PhD^1^; Eiji Nakatani, PhD^2,3^; Midori Tokushima, MD^1^; Akiko Dogomori, MD^1^; Kazumi Mori, MD^1^; Shun Yamashita, MD^1^; Yoshimasa Oda, MD^4^; Shu-ichi Yamashita, MD, PhD^1^

^1^Department of General Medicine, Saga University Hospital, Saga, Japan

^2^Graduate School of Public Health, Shizuoka Graduate University of Public Health, Shizuoka, Japan

^3^Translational Research Center for Medical Innovation, Foundation for Biomedical Research and Innovation at Kobe, Hyogo, Japan

^4^Department of General Medicine, Yuai-Kai Foundation and Oda Hospital, Saga, Japan

**Corresponding author:** Masaki Tago, Department of General Medicine, Saga University Hospital, Saga, Japan. Address: 5-1-1 Nabeshima, Saga, 849-8501 Japan. TEL: +81-952-34-3238. FAX: +81-952-34-2029. E-mail: [tagomas@cc.saga-u.ac.jp](mailto:tagomas@cc.saga-u.ac.jp)

**Supporting Information file**

**S1, Appendix. Characteristics of the two hospitals**

**Yuai-Kai Foundation and Oda Hospital: Hospital O**

The hospital contains 11 departments with 111 beds for acute care. The departments are Internal Medicine, General Medicine, General Surgery, Cardiovascular Surgery, Neurosurgery, Otorhinolaryngology, Plastic Surgery, Dermatology, Radiology, Anesthesiology, and Rehabilitation. It has no Orthopedic Surgery department. The hospital is located in the city of Kashima, in Saga prefecture in southern Japan. It covers an approximate population of 90,000, and treats approximately 3,100 inpatients each year, with a mean length of stay of 12.1 days.

**Saga City Fuji-Yamato Spa Hospital: Hospital F**

The hospital contains five departments with 98 beds, of which 54 are for acute care and 44 for chronic care. Its departments are Internal Medicine, General Medicine, General Surgery, Orthopedic Surgery, and Rehabilitation. The hospital is located in a rural area, Fuji, in Saga prefecture. It treats over 650 inpatients each year, with a mean length of stay of 19.1 days for acute care beds and 71.3 days for chronic care beds.
