# Supplementary material for: External validation of a new predictive model for falls among inpatients using the official Japanese ADL scale, Bedriddenness ranks: a double-centered prospective cohort study
Source: BMC Geriatr. 2022 Apr 15;22:331. doi: 10.1186/s12877-022-02871-5 (PMC9013105; doi:10.1186/s12877-022-02871-5)
Supplement: Supplementary file 3 — Additional file 3: Appendix S3. The formula of model 2. [file 12877_2022_2871_MOESM3_ESM.docx]

External validation of a new predictive model for falls among inpatients using the official Japanese ADL scale, Bedriddenness ranks: A double-centered prospective cohort study

Masaki Tago, MD, PhD^1^*; Naoko E. Katsuki, MD, PhD^1^; Eiji Nakatani, PhD^2,3^; Midori Tokushima, MD^1^; Akiko Dogomori, MD^1^; Kazumi Mori, MD^1^; Shun Yamashita, MD^1^; Yoshimasa Oda, MD^4^; Shu-ichi Yamashita, MD, PhD^1^

^1^Department of General Medicine, Saga University Hospital, Saga, Japan

^2^Graduate School of Public Health, Shizuoka Graduate University of Public Health, Shizuoka, Japan

^3^Translational Research Center for Medical Innovation, Foundation for Biomedical Research and Innovation at Kobe, Hyogo, Japan

^4^Department of General Medicine, Yuai-Kai Foundation and Oda Hospital, Saga, Japan

**Corresponding author:** Masaki Tago, Department of General Medicine, Saga University Hospital, Saga, Japan. Address: 5-1-1 Nabeshima, Saga, 849-8501 Japan. TEL: +81-952-34-3238. FAX: +81-952-34-2029. E-mail: [tagomas@cc.saga-u.ac.jp](mailto:tagomas@cc.saga-u.ac.jp)

**Supporting Information file**

**S3, Appendix. The formula of model 2.**

**Formula of model 2**

−5.8563 + 0.0096 × (Age) + (Male = 0.5684) + (Emergency admission = 0.4418) + (Admitted department; Neurosurgery = 0.6520) + (Hypnotics; Using = 0.2139, Missing data = 0.3612) + (History of fall = 0.4362) + (Ability of eating; Independent = 0.2352, Missing data = −1.0436) + (Bedriddenness rank; J = 1.3758, A = 1.8317, B = 1.9186, C = 1.7205, Not assessable = −0.1505).
