# Supplementary material for: External validation of a new predictive model for falls among inpatients using the official Japanese ADL scale, Bedriddenness ranks: a double-centered prospective cohort study
Source: BMC Geriatr. 2022 Apr 15;22:331. doi: 10.1186/s12877-022-02871-5 (PMC9013105; doi:10.1186/s12877-022-02871-5)
Supplement: Supplementary file 4 — Additional file 4: Figure S4. The incidence of falls among inpatients, by age group [file 12877_2022_2871_MOESM4_ESM.pptx]

## Slide 1
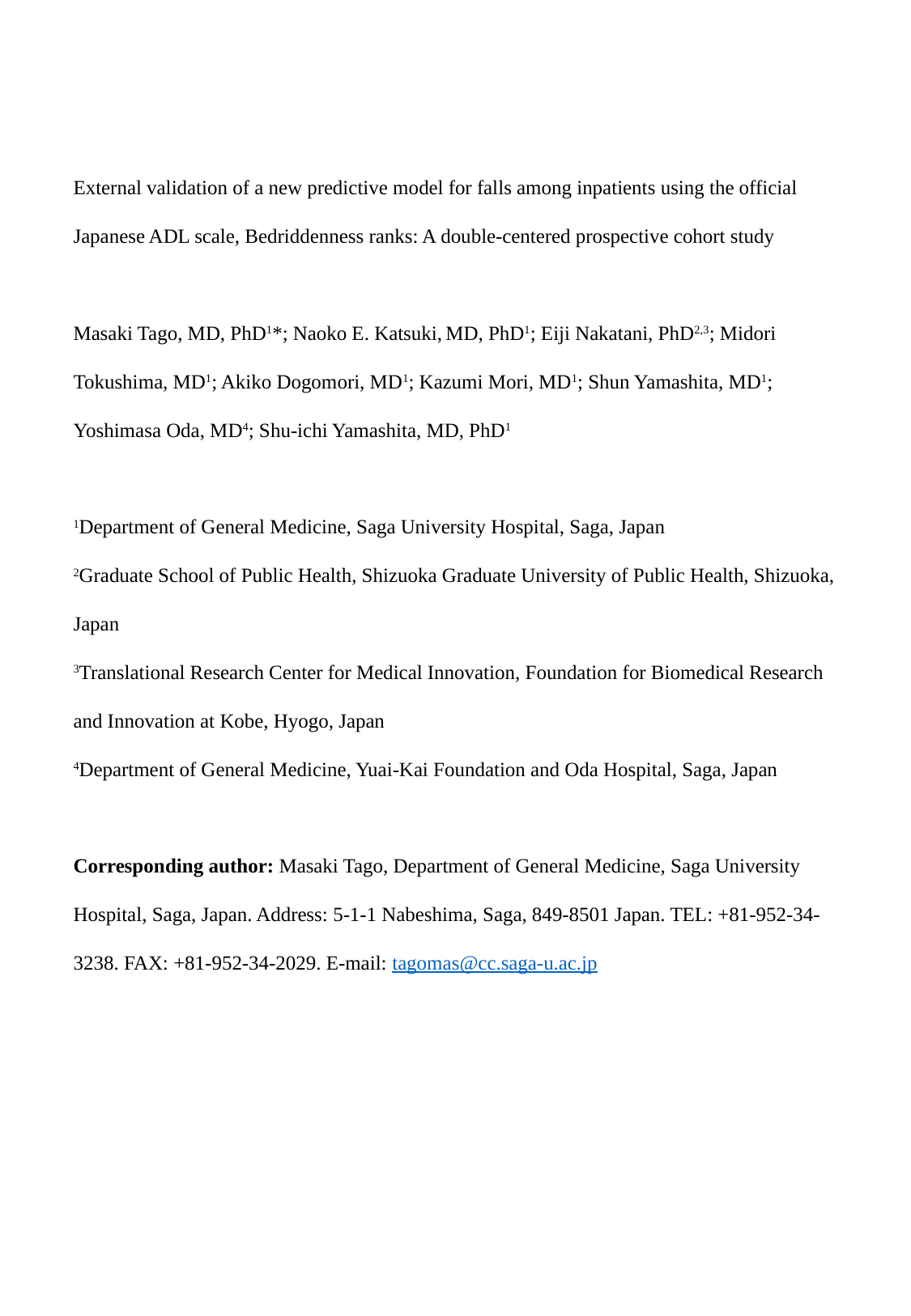

External validation of a new predictive model for falls among inpatients using the official Japanese ADL scale, Bedriddenness ranks: A double-centered prospective cohort study
Masaki Tago, MD, PhD1*; Naoko E. Katsuki, MD, PhD1; Eiji Nakatani, PhD2,3; Midori Tokushima, MD1; Akiko Dogomori, MD1; Kazumi Mori, MD1; Shun Yamashita, MD1; Yoshimasa Oda, MD4; Shu-ichi Yamashita, MD, PhD1
1Department of General Medicine, Saga University Hospital, Saga, Japan
2Graduate School of Public Health, Shizuoka Graduate University of Public Health, Shizuoka, Japan
3Translational Research Center for Medical Innovation, Foundation for Biomedical Research and Innovation at Kobe, Hyogo, Japan
4Department of General Medicine, Yuai-Kai Foundation and Oda Hospital, Saga, Japan
Corresponding author: Masaki Tago, Department of General Medicine, Saga University Hospital, Saga, Japan. Address: 5-1-1 Nabeshima, Saga, 849-8501 Japan. TEL: +81-952-34-3238. FAX: +81-952-34-2029. E-mail: tagomas@cc.saga-u.ac.jp

## Slide 2
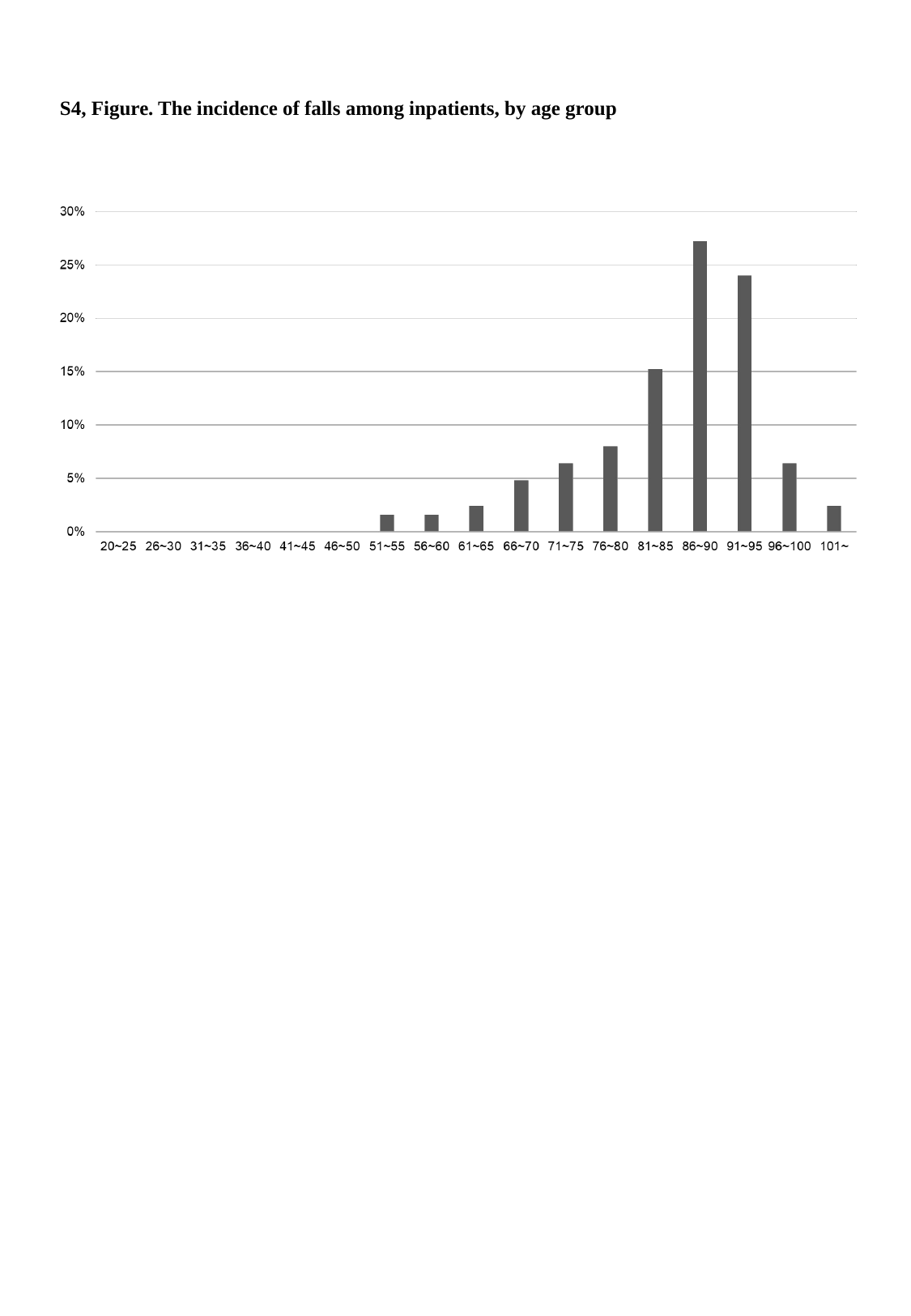

S4, Figure. The incidence of falls among inpatients, by age group
