# Supplementary material for: External validation of a new predictive model for falls among inpatients using the official Japanese ADL scale, Bedriddenness ranks: a double-centered prospective cohort study
Source: BMC Geriatr. 2022 Apr 15;22:331. doi: 10.1186/s12877-022-02871-5 (PMC9013105; doi:10.1186/s12877-022-02871-5)
Supplement: Supplementary file 5 — Additional file 5: Table S5. Classification of primary condition causing emergency or scheduled admission to hospital. [file 12877_2022_2871_MOESM5_ESM.docx]

External validation of a new predictive model for falls among inpatients using the official Japanese ADL scale, Bedriddenness ranks: A double-centered prospective cohort study

Masaki Tago, MD, PhD^1^*; Naoko E. Katsuki, MD, PhD^1^; Eiji Nakatani, PhD^2,3^; Midori Tokushima, MD^1^; Akiko Dogomori, MD^1^; Kazumi Mori, MD^1^; Shun Yamashita, MD^1^; Yoshimasa Oda, MD^4^; Shu-ichi Yamashita, MD, PhD^1^

^1^Department of General Medicine, Saga University Hospital, Saga, Japan

^2^Graduate School of Public Health, Shizuoka Graduate University of Public Health, Shizuoka, Japan

^3^Translational Research Center for Medical Innovation, Foundation for Biomedical Research and Innovation at Kobe, Hyogo, Japan

^4^Department of General Medicine, Yuai-Kai Foundation and Oda Hospital, Saga, Japan

**Corresponding author:** Masaki Tago, Department of General Medicine, Saga University Hospital, Saga, Japan. Address: 5-1-1 Nabeshima, Saga, 849-8501 Japan. TEL: +81-952-34-3238. FAX: +81-952-34-2029. E-mail: [tagomas@cc.saga-u.ac.jp](mailto:tagomas@cc.saga-u.ac.jp)

**Supporting Information file**

**S5, Table. Classification of primary condition causing emergency or scheduled admission to hospital**

| **Hospital O** | | | **Hospital F** | | |
| --- | --- | --- | --- | --- | --- |
| **Emergency admission** | | | | | |
| **Disease** | **n^†^** | **%** | **Disease** | **n^‡^** | **%** |
| Acute pneumonia | 58 | 6.5 | Aspiration pneumonia | 46 | 9.7 |
| Aspiration pneumonia | 58 | 6.5 | Acute pneumonia | 35 | 7.4 |
| Congestive heart failure | 55 | 6.1 | Acute exacerbation of chronic heart failure | 20 | 4.2 |
| Chronic heart failure | 34 | 3.8 | Acute pyelonephritis | 20 | 4.2 |
| Urinary tract infection | 31 | 3.4 | Dehydration | 16 | 3.4 |
| Bronchial pneumonia | 27 | 3.0 | Acute biliary duct infection | 12 | 2.5 |
| Acute pyelonephritis | 27 | 3.0 | Chronic renal failure | 10 | 2.1 |
| Acute atherothrombotic stroke | 20 | 2.2 | Congestive heart failure | 8 | 1.7 |
| Acute ischemic colitis | 14 | 1.6 | Acute obstructive suppurative cholangitis | 8 | 1.7 |
| Dehydration | 14 | 1.6 | Chronic heart failure | 7 | 1.5 |
| **Scheduled admission** | | | | | |
| **Disease** | **n^§^** | **%** | **Disease** | **n^⁋^** | **%** |
| Herpes zoster | 111 | 5.4 | Colorectal polyp | 7 | 6.6 |
| Exertional angina pectoris | 82 | 4.0 | Trochanteric fracture of the femur | 6 | 5.7 |
| Non-Hodgkin's lymphoma | 52 | 2.5 | Aspiration pneumonia | 5 | 4.7 |
| Acute pneumonia | 41 | 2.0 | Complications to permanent residual damage from previous stroke | 5 | 4.7 |
| Congestive heart failure | 38 | 1.8 | Acute pneumonia | 4 | 3.8 |
| Epidermoid cyst | 37 | 1.8 | Atrophic Lateral Sclerosis | 2 | 1.9 |
| Inguinal hernia | 37 | 1.8 | Pelvic fracture | 2 | 1.9 |
| Aspiration pneumonia | 36 | 1.7 | Sequelae of cerebellar hemorrhage | 2 | 1.9 |
| Bronchial pneumonia | 36 | 1.7 | Ascending colon cancer | 2 | 1.9 |
| Benign Paroxysmal Positional Vertigo | 24 | 1.2 | Pyelonephritis | 2 | 1.9 |

† n = 899, ‡ n = 475, § n = 2,071, ⁋ n = 106
