# Supplementary material for: External validation of a new predictive model for falls among inpatients using the official Japanese ADL scale, Bedriddenness ranks: a double-centered prospective cohort study
Source: BMC Geriatr. 2022 Apr 15;22:331. doi: 10.1186/s12877-022-02871-5 (PMC9013105; doi:10.1186/s12877-022-02871-5)
Supplement: Supplementary file 6 — Additional file 6: Table S6. Validation of the predictive model with the cutoff points of the previous study. [file 12877_2022_2871_MOESM6_ESM.docx]

External validation of a new predictive model for falls among inpatients using the official Japanese ADL scale, Bedriddenness ranks: A double-centered prospective cohort study

Masaki Tago, MD, PhD^1^*; Naoko E. Katsuki, MD, PhD^1^; Eiji Nakatani, PhD^2,3^; Midori Tokushima, MD^1^; Akiko Dogomori, MD^1^; Kazumi Mori, MD^1^; Shun Yamashita, MD^1^; Yoshimasa Oda, MD^4^; Shu-ichi Yamashita, MD, PhD^1^

^1^Department of General Medicine, Saga University Hospital, Saga, Japan

^2^Graduate School of Public Health, Shizuoka Graduate University of Public Health, Shizuoka, Japan

^3^Translational Research Center for Medical Innovation, Foundation for Biomedical Research and Innovation at Kobe, Hyogo, Japan

^4^Department of General Medicine, Yuai-Kai Foundation and Oda Hospital, Saga, Japan

**Corresponding author:** Masaki Tago, Department of General Medicine, Saga University Hospital, Saga, Japan. Address: 5-1-1 Nabeshima, Saga, 849-8501 Japan. TEL: +81-952-34-3238. FAX: +81-952-34-2029. E-mail: [tagomas@cc.saga-u.ac.jp](mailto:tagomas@cc.saga-u.ac.jp)

**S6, Table. Validation of the predictive model with the cutoff points of the previous study**

| Statistics for 3 cutoff points | Overall | Hospital O | Hospital F |
| --- | --- | --- | --- |
| Cutoff value for scores | −3.80 | −3.80 | −3.80 |
| Probability^†^ | 2.2 | 2.2 | 2.2 |
| Sensitivity | 91 | 93 | 88 |
| Specificity | 50 | 56 | 19 |
| Positive predictive value | 6.2 | 5.8 | 8.2 |
| Negative predictive value | 99 | 100 | 95 |
| Cutoff value for scores | −2.78 | −2.78 | −2.78 |
| Probability^†^ | 5.9 | 5.9 | 5.9 |
| Sensitivity | 72 | 70 | 76 |
| Specificity | 71 | 76 | 39 |
| Positive predictive value | 8.2 | 8.0 | 9.3 |
| Negative predictive value | 99 | 99 | 95 |
| Cutoff value for scores | −2.01 | −2.01 | −2.01 |
| Probability^†^ | 11.8 | 11.8 | 11.8 |
| Sensitivity | 27 | 24 | 32 |
| Specificity | 94 | 95 | 86 |
| Positive predictive value | 13.3 | 12.4 | 15.3 |
| Negative predictive value | 97 | 98 | 94 |

^†^The value was calculated as the probability of a fall for patients with defined score.
