# Supplementary material for: External validation of a new predictive model for falls among inpatients using the official Japanese ADL scale, Bedriddenness ranks: a double-centered prospective cohort study
Source: BMC Geriatr. 2022 Apr 15;22:331. doi: 10.1186/s12877-022-02871-5 (PMC9013105; doi:10.1186/s12877-022-02871-5)
Supplement: Supplementary file 8 — Additional file 8: Table S8. Patient backgrounds and results of univariate analysis by hospital. [file 12877_2022_2871_MOESM8_ESM.docx]

External validation of a new predictive model for falls among inpatients using the official Japanese ADL scale, Bedriddenness ranks: A double-centered prospective cohort study

Masaki Tago, MD, PhD^1^*; Naoko E. Katsuki, MD, PhD^1^; Eiji Nakatani, PhD^2,3^; Midori Tokushima, MD^1^; Akiko Dogomori, MD^1^; Kazumi Mori, MD^1^; Shun Yamashita, MD^1^; Yoshimasa Oda, MD^4^; Shu-ichi Yamashita, MD, PhD^1^

^1^Department of General Medicine, Saga University Hospital, Saga, Japan

^2^Graduate School of Public Health, Shizuoka Graduate University of Public Health, Shizuoka, Japan

^3^Translational Research Center for Medical Innovation, Foundation for Biomedical Research and Innovation at Kobe, Hyogo, Japan

^4^Department of General Medicine, Yuai-Kai Foundation and Oda Hospital, Saga, Japan

**Corresponding author:** Masaki Tago, Department of General Medicine, Saga University Hospital, Saga, Japan. Address: 5-1-1 Nabeshima, Saga, 849-8501 Japan. TEL: +81-952-34-3238. FAX: +81-952-34-2029. E-mail: [tagomas@cc.saga-u.ac.jp](mailto:tagomas@cc.saga-u.ac.jp)

**Supporting Information file**

**S8, Table.** Patient backgrounds and results of univariate analysis by hospital

| Variable, Category | Hospital O | | | | Hospital F | | | |
| --- | --- | --- | --- | --- | --- | --- | --- | --- |
|  | All | With Fall | Without fall | *p* value^†^ | All | With Fall | Without fall | *p* value^†^ |
|  | n=2,970 | n=84 | n=2,886 |  | n=581 | n=41 | n=540 |  |
| Age, years | 77 (64-86) | 87 (79-91) | 76 (63-85) | <0.001 | 84 (74-90) | 85 (80-89) | 84 (73-90) | 0.527 |
| Gender, Male | 1,431 (48.2) | 40 (47.6) | 1,391 (48.2) | 0.917 | 270 (46.5) | 19 (46.3) | 251 (46.5) | 0.986 |
| Emergency admission, Yes | 899 (30.3) | 45 (53.6) | 854 (29.6) | <0.001 | 475 (81.8) | 32 (78.0) | 443 (82.0) | 0.524 |
| Transported by ambulance, Yes | 389 (13.1) | 10 (11.9) | 379 (13.1) | 0.742 | 84 (14.5) | 5 (12.2) | 79 (14.6) | 0.669 |
| Referral letter, Presence | 1,027 (34.6) | 38 (45.2) | 989 (34.3) | 0.037 | 37 (6.4) | 5 (12.2) | 32 (5.9) | 0.113 |
| Department, Internal Medicine | 1,475 (49.7) | 56 (66.7) | 1419 (49.2) | <0.001 | 524 (90.2) | 33 (80.5) | 491 (90.9) | 0.030 |
| Department, Neurosurgery | 68 (2.3) | 7 (8.3) | 61 (2.1) |  | NA | NA | NA | NA |
| Hypnotic medications, Using | 250 (8.4) | 18 (21.4) | 232 (8.0) | <0.001 | 107 (18.4) | 10 (24.4) | 97 (18.0) | 0.279 |
| Hypnotic medications, Missing | 2 (0.1) | 0 (0) | 2 (0.1) |  | 22 (3.8) | 0 (0) | 21 (4.1) |  |
| Permanent residual damage from previous stroke, Presence | 128 (4.3) | 6 (7.1) | 122 (4.2) | 0.195 | 99 (17.7) | 5 (12.2) | 94 (18.1) | 0.337 |
| History of falls, Presence | 216 (7.3) | 20 (23.8) | 196 (6.8) | <0.001 | 136 (24.3) | 23 (56.1) | 113 (21.8) | <0.001 |
| Visual impairment, Presence | 879 (29.6) | 41 (48.8) | 838 (29.1) | <0.001 | 256 (73.8) | 20 (80.0) | 236 (73.3) | 0.463 |
| Eating, Independent | 2,328 (78.4) | 37 (44.0) | 2,291 (79.4) | <0.001 | 385 (66.3) | 29 (70.7) | 356 (65.9) | 0.530 |
| Eating, Missing category | 7 (0.2) | 0 (0) | 7 (0.2) |  | 0 (0) | 0 (0) | 0 (0) |  |
| Bedriddenness rank, Normal | 1,663 (56.0) | 6 (7.1) | 1,657 (57.4) |  | 127 (21.9) | 5 (12.2) | 122 (22.6) | 0.314 |
| Bedriddenness rank, J | 235 (7.9) | 4 (4.8) | 231 (8.0) |  | 82 (14.1) | 6 (14.6) | 76 (14.1) |  |
| Bedriddenness rank, A | 315 (10.6) | 17 (20.2) | 298 (10.3) | <0.001 | 95 (16.4) | 8 (19.5) | 87 (16.1) |  |
| Bedriddenness rank, B | 381 (12.8) | 43 (51.2) | 338 (11.7) |  | 136 (23.4) | 14 (34.1) | 122 (22.6) |  |
| Bedriddenness rank, C | 376 (12.7) | 14 (16.7) | 362 (12.5) |  | 141 (24.3) | 8 (19.5) | 133 (24.6) |  |
| Cognitive function score, Normal | 1,966 (66.2) | 12 (14.3) | 1,954 (67.7) | <0.001 | 221 (38.2) | 8 (20.0) | 213 (39.6) | 0.008 |
| Cognitive function score, 1 | 334 (11.2) | 15 (17.9) | 319 (11.1) |  | 89 (15.4) | 9 (22.5) | 80 (14.9) |  |
| Cognitive function score, 2 | 268 (9.0) | 24 (28.6) | 244 (8.5) |  | 73 (12.6) | 11 (27.5) | 62 (11.5) |  |
| Cognitive function score, 3 | 346 (11.6) | 30 (35.7) | 316 (10.9) |  | 107 (18.5) | 5 (12.5) | 102 (19.0) |  |
| Cognitive function score, 4 | 42 (1.4) | 3 (3.6) | 39 (1.4) |  | 77 (13.3) | 5 (12.5) | 72 (13.4) |  |
| Cognitive function score, M | 5 (0.2) | 0 (0) | 5 (0.2) |  | 11 (1.9) | 2 (5.0) | 9 (1.7) |  |
| Cognitive function score, missing | 9 (0.3) | 0 (0) | 9 (0.3) |  | 0 (0) | 0 (0) | 0 (0) |  |
| Barthel index | 100 (65-100) | 50 (35-65) | 100 (70-100) | <0.001 | 55 (10-95) | 50 (13-75) | 58 (6-100) | 0.394 |
| Katz index | 6 (2-6) | 1 (0-2) | 6 (2-6) | <0.001 | 2 (0-6) | 1 (0-5) | 2 (0-6) | 0.310 |
| Surgical operation, Undergone | 991 (33.4) | 13 (15.5) | 978 (33.9) | <0.001 | NA | NA | NA | NA |
| Rehabilitation, Undergone | 1,245 (41.9) | 75 (89.3) | 1,170 (40.5) | <0.001 | 65 (11.2) | 10 (24.4) | 55 (10.2) | 0.005 |
| Length of hospital stay (days) | 9 (4-16) | 27 (17-40) | 9 (4-15) | <0.001 | 17 (8-38) | 59 (25-96) | 16 (8-36) | <0.001 |

Continuous and categorical variables are shown as median (interquartile range) and frequency (percent). Bedriddenness ranks: J, independence/autonomy; A, house-bound; B, chair-bound; C, bed-bound. Cognitive function scores: I, almost independent in daily living with only slight cognitive impairment; II, independent with slight difficulty in daily living or communication under careful overseeing; III, dependent in daily living or communication; IV, dependent in daily living or communication, and requires constant care; M, severe psychological symptoms, troubled behaviors or severe physical disorders requiring specialized medical services.

^†^*p* values were calculated by Mann–Whitney U-test for continuous variables and chi-squared test for categorical variables.
